# “Association between dietary inflammatory index (DII) and risk of irritable bowel syndrome: a case-control study”

**DOI:** 10.1186/s12937-021-00721-5

**Published:** 2021-06-28

**Authors:** Elham Eslampour, Koroush Ghanadi, Vahideh Aghamohammadi, Alireza Moayed Kazemi, Rasool Mohammadi, Farhad Vahid, Amir Abbasnezhad

**Affiliations:** 1 Student Research Committee, Lorestan University of Medical Sciences, Khorramabad, Iran; 2 Department of Internal Medicine, Faculty of Medicine, Lorestan University of Medical Sciences, Khorramabad, Iran; 3 Department of Nutrition, Khalkhal University of Medical Sciences, Khalkhal, Iran; 4 Razi Center for Herbal Medicine Research, Lorestan University of Medical Sciences, Khorramabad, Iran; 5 Department of Epidemiology and Biostatistics, School of Public Health and Nutrition, Lorestan University of Medical Sciences, Khorramabad, Iran; 6 Population Health Department, Nutrition and Health Research Group, Luxembourg Institute of Health, Strassen, Luxembourg; 7 Nutritional Health Research Center, Department of Nutrition, Lorestan University of Medical Sciences, PO Box: 6813833946, Goledasht Blvd, Khorramabad, Iran

**Keywords:** Dietary assessment- inflammation- dietary inflammatory index (DII)- irritable bowel syndrome (IBS)

## Abstract

**Background:**

Pathophysiology of IBS is not well recognized; however, several studies have shown the possible relationship between diet and risk of IBS. We assessed the ability of the dietary inflammatory index (DII) to predict the risk of IBS.

**Methods:**

The subjects were 155 IBS cases and 310 age- and sex-matched healthy controls (aged ≥18 years). The participants were recruited from June, 2019 to March, 2020. IBS was recognized using the Rome IV criteria. DII score was computed based on dietary intake using a 168-item FFQ. The DII score was calculated based on energy-adjusted amounts of nutrients using residual method. Logistic regression models were used to estimate multivariable odds ratios (ORs).

**Results:**

The mean DII score was significantly higher among IBS patients in comparison to healthy controls (0.78 ± 2.22 vs. − 0.39 ± 2.27). In crude model, increase in DII as continuous variable was associated with a significant increase in the risk of IBS (OR (95% CI): 1.26 (1.1–15.38)). Furthermore, the association remained significant even after adjusting for age and sex (OR (95% CI): 1.28 (1.1–17.41)) and after multivariate adjustment (OR (95% CI): 1.38 (1.2–1.56)). In crude, age and sex adjusted and multivariate-adjusted models subjects in fourth quartile of DII had higher OR in comparison to subjects in first quartile.

**Conclusions:**

This study showed a possible positive association between a pro-inflammatory diet and the risk of IBS. Thus, encouraging intake of more anti-inflammatory dietary factors and reducing intake of pro-inflammatory factors may be a strategy for reducing risk of IBS.

**Supplementary Information:**

The online version contains supplementary material available at 10.1186/s12937-021-00721-5.

## Background

Irritable bowel syndrome (IBS) is a most common chronic gastrointestinal (GI) disorder with abdominal pain and changes in bowel habits [1], without any structural, physiological or biochemical abnormalities in the GI tract [2]. IBS affects 5.7 to 34% of population worldwide [3], and 1.1 to 25% of Iranian adults [4]. IBS has three subtypes including constipation predominant (IBS-C), diarrhea-predominant (IBS-D), and mixed/alternating (A-IBS) subtypes [5]. The etiology of IBS is unknown; however, several factors including genetic, gut hypersensitivity, intestinal microbiota, low-grade mucosal inflammation, disturbed colonic motility, previous GI infection, and disturbances in the gut neuroendocrine system (NES) and dietary factors might be involved in the pathogenesis of this disorder [6]. Chronic inflammation is associated with chronic diseases and IBS patients have been shown to have a low-grade systemic inflammation [7].

Diet is considered to be a key factor in the pathophysiology of IBS and may contribute in the onset or exacerbation of symptoms [8]. It has been shown that dietary factors stimulating inflammatory process might be involved in the IBS pathology [9]. Assessing the overall quality of the diet has been suggested as a potential approach for determining the association between disease and diet [10–12]. One of the most important factors in nutritional evaluation is to assess the anti- or pro-inflammatory properties of a diet, which has recently been evaluated using Dietary Inflammatory Index (DII) [13]. Its validity was demonstrated using various inflammatory markers, including C-reactive protein, interleukin-6, and homocysteine [14]. Chronic pro-inflammatory conditions including obesity, metabolic syndrome and cardiovascular diseases have been extensively linked with DII [15–17]. According to the evidence, there are limited studies on the association between nutrient-based DII and risk of IBS. In a cross-sectional study, consumption of a pro-inflammatory diet was associated with increased odds of IBS, particularly among women and in overweight/obese subjects [9]. In the present study, we aimed to investigate the relationship between DII scores and risk of IBS, and potential relationships between this index and severity of symptoms in IBS patients.

## Materials and methods

### Participants

This case-control study was conducted at several outpatient clinics in khorramabad city of Iran, from June, 2019 to March, 2020. Cases were newly diagnosed IBS patients who were recruited according to the ROME IV criteria by two gastroenterologists [18]. The control group was randomly selected from other caregivers of patients who referred to the clinic with patients and did not have any disease. Along with the IBS patients, sex and age matched healthy controls (HCs) (confirmed by a physician) were recruited via advertisement from local population. Data on cases and controls were collected at the same time and both were interviewed in the same setting. After providing written and verbal explanations about the methodology of the study, informed consent was received from all participants. The sample size was calculated 155 subjects with IBS using the formula of $n=\frac{{\left({z}_{1-\frac{a}{2}}+{z}_{1-\beta}\right)}^2\left({\delta}_1^2+{\delta}_2^2\right)}{{\left({\mu}_1-{\mu}_2\right)}^2}$, based on statistical data of Jalali et al. [19] study follow as α =0.01 and power of the test 90%, μ 1 = 82.65, μ 2 = 89.53, δ1 = 11.71, δ2 = 16.65, Z (1-α/2) = 2.57 and Z (1-β) = 1.28. Also, for each patient, two age and sex matching controls considered. The study protocol was approved by the local Ethics Review Committee at Lorestan University of Medical Sciences

### Inclusion and exclusion criteria

Inclusion criteria for this study were as follow: a) adults (aged ≥18 years), b) be newly diagnosed cases of IBS patients. Exclusion criteria were as follow: a) non-adherence to the study protocol, b) reporting caloric intake > 5500 or < 800 kcal/day, c) severe lethargy, d) patient’s inability to respond to the questions, e) any evidence of abdominal surgery or radiation, celiac disease, or other primary GI illnesses, f) GI infection obscuring IBS symptoms, g) pregnancy or lactation, h) following a special diets such as vegetarian, weight lost or weight gain during the year prior to the interview. Anthropometric and demographic data including age, sex, marital status, weight, height, BMI, occupation, education status, smoking and passive smoking status were collected from participants by self-administered questionnaires.

### Assessment of dietary intake

Dietary intakes of all participants during the past year were collected using a valid and reliable semi-quantitative 168-food item food frequency questionnaire (FFQ) [20]. Frequency of consumption of each food item on a daily, weekly, monthly, or yearly basis were asked from subjects, then, the intake frequencies converted to grams of food intake per day. For calculation of DII, some additional items including turmeric, saffron, black pepper, ginger, rosemary, and thyme were added to FFQ [19]. The dietary data were analyzed by Nutritionist ІV software [21].

### Assessment of DII scores

We used FFQ-derived dietary data for calculating DII scores for all study participants. The DII is based on literature published through 2010 according to the daily intake of food items affecting the profile of inflammation. Individuals’ intakes of food parameters on which the DII is based are then compared to a world standard database (Eleven food consumption data sets from around the world were identified that represent a range of human dietary intakes that serve as the ‘referent’ population database to provide comparative consumption data for these food parameters [13]). A complete description of the DII is available elsewhere [13]. At first, we calculated energy- adjusted amounts of food parameters using residual method. Then, to calculate DII score for each participant, we calculated the z score for a given food parameter by subtracting the “standard global mean” from the amount consumed by each subject and dividing this value by the “global standard deviation”. Global means and standard deviations were obtained from the study of Shivappa et al. [13]. To minimize the effect of ‘right skewing’ (a common occurrence with dietary data), these z scores were then converted to a centered percentile score [13]. The centered percentile score of each food parameter for each subject was then multiplied by the respective effect score of food parameters derived from the study of Shivappa et al. [13], to obtain the food parameter-specific DII score for a subject. All food parameter-specific DII scores were then summed to create the overall DII score for each subject in the study. A higher DII score (more positive) indicates a more pro-inflammatory diet and a lower DII score (more negative) indicates a more anti-inflammatory diet. In the current study, we used 36 items including: energy, carbohydrate, protein, total fat, fiber, cholesterol, SFA, MUFA,PUFA, n-3, n-6, B vitamins, folic acid, Fe, Zn, mg, Se, vitamin A, vitamin C, vitamin D, vitamin E, b-carotene, onion, garlic, caffeine, tea, saffron, ginger, turmeric, pepper, rosemary and thyme.

### Assessment of other variables

The required information of all participants including age (year), sex (male/female), occupation (employee, unemployed, retired, housewife), marital status (single/ married), education (illiterate/ under diploma/ diploma/ upper diploma), smoking status (yes/no), passive smoking status (yes/no), physical activity level (low/ moderate/ severe), anxiety level (mild/ moderate/ severe) and depression level (mild/ moderate/ severe) were obtained by questionnaires. Hospital anxiety and depression scale (HADS) was used to assess both anxiety and depression. The HADS includes fourteen items containing two subscales that seven of these items are associated with anxiety (HADS-A) and the remaining seven with depression (HADS-D). Each item is scored on a 4-points Likert scale ranging from 0 to 3 and the score of each subscale ranging from 0 to 21. Higher scores indicate a worse condition. Scores of 8–10, 11–14 and 15–21 indicate mild, moderate and severe disorders, respectively. In the present study, the Persian version of HADS which has been approved for its validity and reliability was used [22].

International physical activity questionnaire (IPAQ) was used to calculate physical activity of participants, which has already been approved in Iran for its validity and reliability. In terms of MET (metabolic equivalent minutes) and according to the standard protocol, the classification of physical activity were as follow: low activity (below 600 MET-minutes/week), moderate (between 600 and 3000 MET-minutes/week) intense activity (above 3000 MET-minutes/week or at least 1500 MET minutes/week of intensity activity) [23].

The weight of each subject was measured with the least clothes using a SECA digital scale, which is accurate to 100 g. Height was measured without shoes in standing position, leaning against the wall and shoulder blades under normal circumstances with an accuracy of 0.5 cm by the mean of a tape mounted on the wall. Body mass index (BMI) was calculated by dividing weight (in kilograms) by the square of height (square meters). Body Mass Index (BMI) was calculated by dividing weight (kg) to square of height (m) and was categorised into normal weight (BMI < 25·0 kg/m2), overweight (25·0 kg/m2 ≤ BMI < 30·0 kg/m2) and obese (BMI ≥ 30·0 kg/m2). Waist circumference was measured between the lowest rib and the iliac crest. Circumference measurement accuracy was 0.1 cm.

IBS-symptom severity scale (IBS-SSS) was used to investigate IBS symptoms severity. This questionnaire consists of 5 questions using the VAS scale: severity and duration of abdominal pain, severity of abdominal distension, dissatisfaction with bowel habits and interference with life in general quality of life over the past 10 days. Each questions generate a maximum score of 100, so the total score ranges from 0 to 500 with higher scores indicating more severe disease. Mild, moderate and severe cases were determined by scores of 75 to 175, 175 to 300 and > 300 respectively [24].

To evaluate extra-intestinal Symptoms, the Extra-Intestinal Symptoms Severity Scale (EISSS) was used. This questionnaire contains of 15 items including: nausea/vomiting, early satiety, headache, backache, fatigue, excessive belching, excessive bloating, heartburn, urgency for defecate, strain to defecate, feeling of incomplete defecation, urgency for urination, thigh pain, muscles and joints pain, Postprandial fullness. The items are rated by 7-point Likert responses (never = 0 to always = 6. Final score of questionnaire are converted into 0–100 points which higher scores indicating greater severity. In this study the Persian version of EISSS was used [25].

IBS patients completed the Persian version of the IBS-OQL questionnaire, which consists of 34 items with 5 points Likert response scales ranging from 1 to 5 and 8 different subscales as follows: dysphoria, interference with activity, body image, health worry, food avoidance, social reaction, sexual concerns and relationships. Raw scores are transformed into 0–100 points which higher scores indicating better QOL [26].

Trained nutritionists administered all the questionnaires through face-to-face interviews at the clinic when the participants were recruited and asked them to report needed data about each questionnaire*.*

### Statistical analysis

The Kolmogorov–Smirnoff test was used to determine the distribution of data related to normality. The DII was analyses both as a continuous and as a categorical variable. The DII (as quartiles) was examined using the ANOVA test. Mann–Whitney U test or Kruskal–Wallis test was used for variables that were not normally distributed. Moreover, the chi-square test was used for comparing categorical variables. Binary logistic regression was used to estimate odds ratios (ORs) and 95% confidence intervals (CIs). Statistical analyses were performed using SPSSV22 software and all *P* values were based on two sided tests. *P* < 0.05 was considered statistically significant.

## Results

Table 1 shows the distribution of 155 cases of IBS were enrolled out of 230 potentially eligible patients and 310 controls out of 360 potentially eligible subjects, according to selected variables. The cases of IBS had higher DII, waist Circumference, BMI, anxiety and depression levels and history of smoking and passive smoking. However, only DII, passive smoking, anxiety and depression levels were significantly different between groups (Table 1). The controls had higher total energy intake and physical activity level; however, the results were not significant except for energy intake (Table 1). As Table 2 shows, participants in both case and control groups had higher BMI, waist circumference, energy intake and physical activity in fourth quartile versus the first quartile of the DII. However, the results were not significant except for energy intake and physical activity.

**Table 1:** Distribution of 155 irritable bowel syndrome cases and 310 controls according to selected variables

Variables	Mean ± SD or N (%)		
	Cases (*n* = 155)	Controls (*n* = 310)	*P*-value
Age (years)	39.23 ± 12.18	39.23 ± 12.18	1.00
Body mass index (BMI, kg/m2)	25.27 ± 3.72	25.08 ± 3.48	0.945
Waist circumference (WC,cm)	94.15 ± 12.20	92.35 ± 11.47	0.119
Total energy (Kcal)	2231.83 ± 381.51	2321.79 ± 437.27	0.023
Dietary inflammatory index (DII)	0.78 ± 2.22	-0.39 ± 2.27	< 0.001
Sex ^a^			
Females	88 (57%)	176 (57%)	1.00
Males	67 (43%)	134 (43%)	
Education ^a^			
Unlettered	7 (5%)	9 (3%)	0.392
Diploma	27 (17%)	46 (14%)	
Under diploma	33 (21%)	86 (28%)	
Above diploma	88 (57%)	169 (55%)	
Occupation ^a^			
Freelance	59 (38%)	167 (54%)	0.006
Employee	48 (31%)	61 (20%)	
Unemployed	5 (3%)	13 (4%)	
Housewife	43 (28%)	69 (22%)	
Smoking ^a^			
Yes	15 (10%)	19 (6%)	0.187
No	140 (90%)	291 (94%)	
Passive smoking ^a^			
Yes	35 (23%)	44 (14%)	0.026
No	120 (77%)	266 (86%)	
Anxiety level ^a^			
Mild	82 (53%)	277 (89%)	< 0.001
Moderate	33 (21%)	21 (7%)	
Severe	40 (26%)	12 (4%)	
Depression level ^a^			
Mild	89 (57%)	277 (89%)	< 0.001
Moderate	26 (17%)	19 (6%)	
Severe	40 (26%)	14 (5%)	
Physical activity ^a^			
Low activity	92 (59%)	122 (39%)	< 0.001
Moderate activity	43 (28%)	159 (51%)	
Severe activity	20 (13%)	29 (10%)	

All data are shown as mean ± SD, and analyzed by two-sample *t*-test unless otherwise indicated

^a^Data are numbers (%), and were analyzed by chi-squared test or Fisher’s exact test

**Table 2:** Distribution of 155 case and 310 control characteristics across categories of Dietary Inflammatory Index (DII)

Variables			Mean ± SD or N (%)				*P* value
			Quartile1 (<−1.49)	Quartile2 (−1.49 ≤ to < 0.11)	Quartile3 (0.11 ≤ to < 1.65)	Quartile4 (1.65≤)	
Age (year) ^a^		Case	38.85 ± 13.72	37.57 ± 11.66	39.92 ± 13.81	39.72 ± 10.57	0.857
		Control	39.92 ± 12.67	39.75 ± 12.04	38.11 ± 12.26	38.87 ± 11.61	0.771
Body mass index (BMI, kg/m2) ^a^		Case	24.51 ± 2.62	24.52 ± 3.39	25.66 ± 3.83	25.69 ± 4.19	0.330
		Control	24.38 ± 3.68	25.09 ± 3.57	25.52 ± 3.45	25.58 ± 2.90	0.107
Waist circumference (WC,cm) ^a^		Case	89.85 ± 12.01	92.75 ± 11.90	94.47 ± 11.68	96.60 ± 12.47	0.106
		Control	90.05 ± 12.22	92.80 ± 11.83	92.89 ± 11.20	94.55 ± 9.64	0.109
Total energy (Kcal) ^a^		Case	1896.10 ± 360.78	2098.27 ± 298.56	2275.73 ± 349.66	2420.80 ± 322.16	0.000
		Control	2221.55 ± 424.37	2261.26 ± 401 ± 19	2400.40 ± 415.39	2464.10 ± 487.91	0.002
Physical activity (MET) ^a^		Case	782.48 ± 1232.04	710.92 ± 887.50	842.56 ± 1482.24	1676.19 ± 2099.93	0.015
		Control	1060.17 ± 1729.62	1194.27 ± 1335.09	1236.95 ± 1314.58	2028.97 ± 3288.18	0.022
Marital status ^b^	Single	Control	23 (30.7%)	19 (25.3%)	17 (22.7%)	16 (21.3%)	0.849
	Married	Control	67 (28.5%)	67 (28.5%)	59 (25.1%)	42 (17.95)	
	Single	Case	11 (25.6%)	14 (32.6%)	8 (18.6%)	10 (23.3%)	0.003
	Married	Case	16 (14.3%)	14 (12.5%)	34 (30.4%)	48 (42.9%)	
Occupation ^b^	Freelance	Control	48 (28.7%)	45 (26.9%)	38 (22.8%)	36 (21.6%)	0.787
	Employee	Control	20 (32.8%)	17 (27.9%)	13 (21.3%)	11 (18.0%)	
	Unemployed	Control	5 (38.5%)	4 (30.8%)	3 (23.1%)	1 (7.7%)	
	Housewife	Control	17 (24.6%)	20 (29.0%)	22 (31.9%)	10 (14.5%)	
	Freelance	Case	10 (16.9%)	9 (15.3%)	12 (20.3%)	28 (47.5%)	0.388
	Employee	Case	7 (14.6%)	9 (18.8%)	13 (27.1%)	19 (39.6%)	
	Unemployed	Case	1 (20.0%)	2 (40.0%)	2 (40.0%)	0 (0.0%)	
	Housewife	Case	9 (20.9%)	8 (18.6%)	15 (34.9%)	11 (25.6%)	
Smoking ^b^	Yes	Control	3 (15.8%)	5 (26.3%)	4 (21.1%)	7 (36.8%)	0.180
	No	Control	87 (29.9%)	81 (27.8%)	72 (24.7%)	51 (17.5%)	
	Yes	Case	1 (6.7%)	3 (20.0%)	5 (33.3%)	6 (40.0%)	0.704
	No	Case	26 (18.6%)	25 (17.9%)	37 (26.4%)	52 (37.1%)	
Passive smoking ^b^	Yes	Control	73 (27.4%)	78 (29.3%)	67 (25.2%)	48 (18.0%)	0.250
	No	Control	17 (38.6%)	8 (18.2%)	9 (20.5%)	10 (22.7%)	
	Yes	Case	22 (18.3%)	20 (16.7%)	30 (25/0%)	48 (40.0%)	0.451
	No	Case	5 (14.3%)	8 (22.9%)	12 (34.3%)	10 (28.6%)	

^a^The One-way analysis of variance (ANOVA) was used for comparison of the variables between DII quartiles

^b^Data are numbers (%), and were analyzed by chi-squared test or Fisher’s exact test

The higher DII score in both case and control groups was significantly associated with higher intakes of energy, total fat, carbohydrates, saturated fats, W6, MUFA, PUFA, niacin and thiamin and lower intakes of β-carotene, niacin, black pepper, thyme and turmeric. Moreover, the higher DII score in case group was significantly associated with higher intakes of SFA, Fe and protein. In control group the highest DII score was significantly associated with lower intakes of folic acid, vitamin C, magnesium, vitamin B6, dietary fiber, ginger and saffron (Supplementary Table 1).

The crude and adjusted ORs for the associations between both continuous and categorical DIIs with risk of IBS are shown in Table 3. In crude model, increasing in DII as continuous variable was associated with a significant increase in the risk of IBS (OR (95% CI): 1.26 (1.1–15.38), *p* < 0.001). Furthermore, the association remained significant even after adjusting for age and sex (OR (95% CI): 1.28 (1.1–17.41)) and after multivariable adjustment (OR (95% CI): 1.38 (1.2–1.56)). Similarly, when analyses were carried out with DII expressed as quartiles, in crude, age and sex adjusted and multivariate-adjusted models, as the quartiles increased, the risk of IBS also increased significantly (P-trend < 0.001 for all the models). In crude model, subjects in fourth quartile of DII had an OR (95% CI) of 3.33 (1.8, 5.85) in comparison to subjects in first quartile. In addition, after adjustment for age and sex, subjects in fourth quartile had an OR of 3.65 (95% CI: 2.37–6.55) in comparison to subjects in first quartile. Also, after multivariate adjustment, in fourth quartile the OR was 5.66 (95% CI: 2.65–12.07) versus the first quartile. So a similar pattern was demonstrated for DII as both continuous and categorical variables.

**Table 3:** Odds ratios and confidence intervals for the association between Dietary Inflammatory Index (DII) and irritable bowel syndrome

Dietary Inflammatory Index (categorical) OR (95% CI)					P-trend	DII (Continuous) OR (95% CI)	*P*-Value
DII	Quartile1 (<−1.49)	Quartile2 (−1.49 ≤ to < 0.11)	Quartile3 (0.11 ≤ to < 1.65)	Quartile4 (1.65≤)			
Crude model	1 (ref.)	1.08 (0.59, 2.19)	1.84 (1.04, 3.26)	3.33 (1.8, 5.85)	< 0.001	1.26 (1.1, 15.38)	< 0.001
Age and sex adjusted model	1 (ref.)	1.11 (0.6, 2.04)	1.91 (1.07, 3.41)	3.65 (2.37, 6.55)	< 0.001	1.28 (1.1, 17.41)	< 0.001
Multivariate-adjusted model ^a^	1 (ref.)	1.30 (0.61, 2.74)	2.34 (1.13, 4.84)	5.66 (2.65, 12.07)	< 0.001	1.38 (1.2, 1.56)	< 0.001

Binary logistic regression was used to estimate odds ratios (ORs) and 95% confidence intervals (CIs)

^a^Adjusted for age, sex, BMI, energy, Smoking, passive smoking, physical activity, marital status, occupation status, Education status, depression, anxiety

The result of Table 4 demonstrated no significant correlation between DII and IBS-QOL, IBS-SSS, IBS-EISSS, abdominal pain, abdominal distension, bloating, rumbling and total gastrointestinal symptoms in IBS patients.

**Table 4:** Associations between the Dietary Inflammatory Index (DII) and IBS-QOL, IBS-SSS, IBS-EISSS, abdominal pain, abdominal distension, bloating, rumbling and total gastrointestinal symptoms in IBS patients

Variables	Mean ± SD or N (%)				P-Trend	Correlation coefficient (r)	*P* value
	Quartile1 (<−1.49)	Quartile2 (− 1.49 ≤ to < 0.11)	Quartile3 (0.11 ≤ to < 1.65)	Quartile4 (1.65≤)			
IBS-QOL	67.29 ± 15.88	64.60 ± 14.21	66.51 ± 13.16	69.87 ± 13.99	0.392	0.046	0.572
IBS-SSS	242.85 ± 112.10	274.21 ± 97.04	226.73 ± 94.63	235.82 ± 105.59	0.275	0.005	0.949
IBS-EISSS	23.22 ± 12.07	26.64 ± 14.66	25.14 ± 10.61	22.79 ± 12.22	0.516	0.087	0.284
abdominal pain	38.44 ± 30.40	45.54 ± 30.02	32.81 ± 28.92	38.17 ± 29.86	0.382	−0.056	0.485
abdominal distension	48.52 ± 29.27	51.32 ± 27.35	45.05 ± 26.33	46.57 ± 29.14	0.818	0.036	0.657
Bloating	45.37 ± 31.32	56.50 ± 32.92	51.98 ± 28.84	48.74 ± 31.36	0.559	0.078	0.337
Rumbling	42.89 ± 35.27	43.89 ± 31.93	41.38 ± 30.66	41.38 ± 30.66	0.985	0.085	0.293
Total gastrointestinal symptoms	55.96 ± 33.06	70.21 ± 27.52	58.64 ± 27.22	59.76 ± 28.34	0.258	−0.021	0.799

*IBS* irritable bowel syndrome, *IBS-QOL* IBS-quality of life, *IBS-SSS* IBS-symptom severity scale, *IBS-EISSS* IBS-extra-intestinal symptom severity

The One-way analysis of variance (ANOVA) was used for comparison of the variables between DII quartiles

## Discussion

In this case-control study among a sample of Iranian population, we found that a greater DII score was significantly associated with increased risk of IBS. However, our results showed no significant correlations between a pro-inflammatory diet and IBS-QOL, IBS-SSS, IBS-EISSS, abdominal pain, abdominal distension, bloating, rumbling, and overall abdominal symptoms in patients with IBS. The results of this study highlight the great importance of preventive or therapeutically strategies in the management of IBS, as one of the major gastrointestinal disorders.

Based on the physio-pathological evidence low-grade systemic inflammation plays a critical role in the pathogenesis of IBS [27], therefore, potential dietary simulants with pro-inflammatory capacity may lead to the incidence and exacerbation of IBS symptoms. There are limited data on the associations between the inflammatory potential of diet, as represent as high DII score, and risk of IBS [9, 28]. Nonetheless, our findings are in accordance with the results of previous research. In a cross-sectional study among the Iranian population, a higher DII score was significantly associated with increased odds of IBS [9]. Another secondary analysis on this cross-sectional study data has been shown that empirically derived food-based dietary inflammatory index, another indicator of the inflammatory potential of diet, was significantly associated with increased odds of IBS, especially in women and in overweight and obese subjects [28]. Although this study was also conducted among the Iranian population, however, this had a cross-sectional design, which due to its nature cannot elucidate causal relationships. The investigators also used a dish-based semi-quantitative FFQ with only 106-items and the final DII score consisted of 29 food parameters that might lead to uncertain score estimation. Moreover, the cases in our study were selected using Rome IV criteria whereas that study used Rome III criteria. Accordingly, several studies have been shown direct associations between increasing DII score and higher circulatory levels of inflammatory markers, as main indicators of systemic inflammation [29–31]. Besides, regarding other inflammation-related conditions, the results of the previous research highlight the role of the inflammatory potential of diet in the development of overweight and obesity [15], metabolic syndrome [16], and ulcerative colitis [32].

As another important finding, we observed that the controls had lower DII scores than the cases and tended to have a higher intake of other macro and micronutrients. These findings are similar to the results of the previous research [33, 34]. Interestingly, these findings reveal the fact that the subjects with lower DII scores may adhere to a nutritionally balanced diet including various foods. Some previous research found that patients with IBS had lower QOL than the general population [35, 36]. Other studies also showed that poor QOL in IBS patients might be due to several factors [37, 38]. Interestingly, there is some evidence regarding the effects of dietary factors on QOL in patients with IBS [39–41]. However, we failed to show a significant correlation between DII score and QOL in IBS patients, which warranted further research to elucidate more certain decisions in this case.

Finally, the results of this study did not show any correlations between the inflammatory potential of diet and IBS severity and symptoms. Our literature review on previous research showed that DII and FDII scores were not also associated with IBS severity in a cross-sectional study of another Iranian population [9, 28]. In contrast, the results of a case-control study in the Netherlands indicated that various foods might contribute to special symptoms severity in IBS patients [8]. In general, on one side, it seems several factors such as geographical and cultural related dietary variations and different methods used to assess dietary habits might contribute to different findings from different studies. On the other side, because of limited data on the associations between inflammatory potential of diet and IBS risk and severity (especially from other countries), further evidence is needed to attain a more comprehensive decision to elucidate the possible role of the inflammatory potential of diet in the pathogenesis of IBS disease.

No well-known mechanisms are indicating the role of a pro-inflammatory diet in increasing the risk of IBS, however, some potential factors may be involved [29, 31, 34]. Previous studies have shown a direct association between DII score and serum levels of pro-inflammatory cytokines [29, 31, 34]. These cytokines could affect cellular signaling pathways and lead to increased peripheral and central hypersensitivity [42]. Other research also showed that increased concentrations of inflammatory cytokines are associated with activation of the immune system as a common concern in IBS patients with diarrhea [43]. Secondly, greater adherence to a pro-inflammatory diet was positively associated with increased risk of obesity [15] which in turn is a risk factor for gastrointestinal transit alterations that might also explain IBS incidence and symptoms [44].

Our study has some strengths. First, this is the first well-conducted case-control study indicating a potential association between DII and risk of IBS. Second, the cases and controls were matched for age and sex and were selected from the same center. Third, we controlled analyses for several confounders to make the results less prone to bias. Forth, dietary data were gathered using a valid and reliable FFQ, which included approximately all food items in the diet of the Iranian population. However, FFQ has several limitations. One of the major limitations of the FFQ is its reliance on the respondent’s memory for collecting information for as far back as 12 months [45]. Other limitations of the FFQ are measurement errors including selective under- or over-reporting of intakes of certain foods and requiring skilled professionals for the interview [45]. Another limitation of our study is that the possibility of other confounding factors that are not measured cannot be neglected. Moreover, we considered 36 food parameters for calculating the DII score due to limited data on other anti or pro-inflammatory parameters. Furthermore, other limitations of this study are the relatively low number of patients for this types of variables (although we presented the way the sample size was calculated), and the study population which is exclusively from Iran, thus can limit the general value of the paper.

## Conclusions

The results of this case-control study showed a possible positive association between a pro-inflammatory diet and the risk of IBS. Thus, encouraging intake of more anti-inflammatory dietary factors and reducing intake of pro-inflammatory factors may be a strategy for reducing risk of some cases of IBS. However, we could not correlate the inflammatory potential of a diet with QOL and IBS symptoms in patients with IBS. Therefore, because of some limitations of this study and limited evidence regarding the associations between DII and IBS risk and IBS-related complications especially from other countries, further studies are needed to confirm these findings.

## Supplementary Information

**Additional file 1.**

## Data Availability

All data and materials generated or analyzed during this study are available upon request to the corresponding author.

## Abbreviations

DII: Dietary Inflammatory Index
IBS: Irritable Bowel Syndrome
IBS-C: IBS-Constipation predominant
IBS-D: IBS-Diarrhea predominant
IBS-A: IBS- alternating
GI: Gastrointestinal
NES: Neuroendocrine system
HCs: Healthy controls
BMI: Body mass index
HADS: Hospital anxiety and depression scale
FFQ: Food frequency questionnaire
SFA: Saturated fatty acids
MUFA: Monounsaturated fatty acids
PUFA: Polyunsaturated fatty acids
IPAQ: Physical activity questionnaire
MET: Metabolic equivalent minutes
IBS-SSS: IBS-symptom severity scale
VAS: Visual Analogue Scale
EISSS: Extra-Intestinal Symptoms Severity Scale
QOL: Quality of life
OR: Odds ratios
CI: Confidence interval
SD: Standard Deviation
WC: Waist Circumference

## References

[1] Holtmann GJ, Ford AC, Talley NJ (2016). Pathophysiology of irritable bowel syndrome. Lancet Gastroenterol Hepatol.

[2] Guo Y-B, Zhuang K-M, Kuang L, Zhan Q, Wang X-F, Liu S-D (2015). Association between diet and lifestyle habits and irritable bowel syndrome: a case-control study. Gut Liver.

[3] Oka P, Parr H, Barberio B, Black CJ, Savarino EV, Ford AC (2020). Global prevalence of irritable bowel syndrome according to Rome III or IV criteria: a systematic review and meta-analysis. Lancet Gastroenterol Hepatol.

[4] Jahangiri P, Jazi MSH, Keshteli AH, Sadeghpour S, Amini E, Adibi P (2012). Irritable bowel syndrome in Iran: SEPAHAN systematic review No. 1. Int J Prev Med.

[5] El-Salhy M (2012). Irritable bowel syndrome: diagnosis and pathogenesis. World J Gastroenterol: WJG.

[6] Lee YJ, Park KS (2014). Irritable bowel syndrome: emerging paradigm in pathophysiology. World J Gastroenterol: WJG.

[7] Ng QX, Soh AYS, Loke W, Lim DY, Yeo WS (2018). The role of inflammation in irritable bowel syndrome (IBS). J Inflamm Res.

[8] Tigchelaar EF, Mujagic Z, Zhernakova A, Hesselink M, Meijboom S, Perenboom C (2017). Habitual diet and diet quality in irritable bowel syndrome: a case-control study. Neurogastroenterol Motil.

[9] Salari-Moghaddam A, Keshteli AH, Esmaillzadeh A, Adibi P (2019). Adherence to the pro-inflammatory diet in relation to prevalence of irritable bowel syndrome. Nutr J.

[10] Abdollahpour I, Jakimovski D, Shivappa N, Hébert JR, Vahid F, Nedjat S, Mansournia MA, Weinstock-Guttman B (2020). Dietary inflammatory index and risk of multiple sclerosis: findings from a large population-based incident case-control study. Clin Nutr.

[11] Kashkooli S, Choghakhori R, Hasanvand A, Abbasnezhad A (2019). Effect of calcium and vitamin D co-supplementation on lipid profile of overweight/obese subjects: a systematic review and meta-analysis of the randomized clinical trials. Obesity Med.

[12] Eslampour E, Ebrahimzadeh F, Abbasnezhad A, Khosroshahi MZ, Choghakhori R, Asbaghi O (2019). Association between circulating irisin and C-reactive protein levels: a systematic review and meta-analysis. Endocrinol Metab.

[13] Shivappa N, Steck SE, Hurley TG, Hussey JR, Hébert JR (2014). Designing and developing a literature-derived, population-based dietary inflammatory index. Public Health Nutr.

[14] Shivappa N, Steck SE, Hurley TG, Hussey JR, Ma Y, Ockene IS, Tabung F, Hébert JR (2014). A population-based dietary inflammatory index predicts levels of C-reactive protein in the seasonal variation of blood cholesterol study (SEASONS). Public Health Nutr.

[15] Ramallal R, Toledo E, Martínez JA, Shivappa N, Hébert JR, Martínez-González MA, Ruiz-Canela M (2017). Inflammatory potential of diet, weight gain, and incidence of overweight/obesity: the SUN cohort. Obesity..

[16] Neufcourt L, Assmann K, Fezeu L, Touvier M, Graffouillère L, Shivappa N (2015). Prospective association between the dietary inflammatory index and metabolic syndrome: findings from the SU. VI. MAX study. Nutr Metab Cardiovasc Dis.

[17] Tyrovolas S, Koyanagi A, Kotsakis GA, Panagiotakos D, Shivappa N, Wirth MD, Hébert JR, Haro JM (2017). Dietary inflammatory potential is linked to cardiovascular disease risk burden in the US adult population. Int J Cardiol.

[18] Drossman DA, Hasler WL (2016). Rome IV—functional GI disorders: disorders of gut-brain interaction. Gastroenterology..

[19] Jalali S, Shivappa N, Hébert JR, Heidari Z, Hekmatdoost A, Rashidkhani B (2018). Dietary inflammatory index and odds of breast cancer in a case-control study from Iran. Nutr Cancer.

[20] Esfahani FH, Asghari G, Mirmiran P, Azizi F (2010). Reproducibility and relative validity of food group intake in a food frequency questionnaire developed for the Tehran lipid and glucose study. J Epidemiol.

[21] Mirmiran P, Esfahani FH, Mehrabi Y, Hedayati M, Azizi F (2010). Reliability and relative validity of an FFQ for nutrients in the Tehran lipid and glucose study. Public Health Nutr.

[22] Amini P, Maroufizadeh S, Omani SR (2017). Evaluating the factor structure, item analyses, and internal consistency of hospital anxiety and depression scale in Iranian infertile patients. Int J Reprod Biomed.

[23] Moghaddam MB, Aghdam FB, Jafarabadi MA, Allahverdipour H, Nikookheslat SD, Safarpour S (2012). The Iranian version of international physical activity questionnaire (IPAQ) in Iran: content and construct validity, factor structure, internal consistency and stability. World Appl Sci J.

[24] Francis CY, Morris J, Whorwell PJ (1997). The irritable bowel severity scoring system: a simple method of monitoring irritable bowel syndrome and its progress. Aliment Pharmacol Ther.

[25] Nemati K, Bagherian R, Kheir-Abadi Gh DH, Davazdah-Emami M, Gholamrezaei A (2010). Coping strategies training in the Management of Irritable Bowel Syndrome. J Isfahan Med School.

[26] Abbasi MHB, Safizadeh H, Zahedi MJ, Moghadam SD, Shafiei-pour S, Rafsanjani AMB (2015). Assessment of quality of life in patients with irritable bowel syndrome: a cross-sectional study in south-East Iran. Govaresh..

[27] Sinagra E, Pompei G, Tomasello G, Cappello F, Morreale GC, Amvrosiadis G, Rossi F, Monte AIL, Rizzo AG, Raimondo D (2016). Inflammation in irritable bowel syndrome: myth or new treatment target?. World J Gastroenterol.

[28] Salari-Moghaddam A, Keshteli AH, Esmaillzadeh A, Adibi P (2019). Empirically derived food-based inflammatory potential of the diet, irritable bowel syndrome, and its severity. Nutrition..

[29] Shivappa N, Hebert JR, Marcos A, Diaz LE, Gomez S, Nova E, Michels N, Arouca A, González-Gil E, Frederic G, González-Gross M, Castillo MJ, Manios Y, Kersting M, Gunter MJ, de Henauw S, Antonios K, Widhalm K, Molnar D, Moreno L, Huybrechts I (2017). Association between dietary inflammatory index and inflammatory markers in the HELENA study. Mol Nutr Food Res.

[30] Shivappa N, Hébert JR, Rietzschel ER, De Buyzere ML, Langlois M, Debruyne E (2015). Associations between dietary inflammatory index and inflammatory markers in the Asklepios study. Br J Nutr.

[31] Yang Y, Hozawa A, Kogure M, Narita A, Hirata T, Nakamura T, Tsuchiya N, Nakaya N, Ninomiya T, Okuda N, Kadota A, Ohkubo T, Okamura T, Ueshima H, Okayama A, Miura K (2020). Dietary inflammatory index positively associated with high-sensitivity C-reactive protein level in Japanese from NIPPON data 2010. J Epidemiol.

[32] Shivappa N, Hébert JR, Rashvand S, Rashidkhani B, Hekmatdoost A (2016). Inflammatory potential of diet and risk of ulcerative colitis in a case–control study from Iran. Nutr Cancer.

[33] Suzuki K, Shivappa N, Kawado M, Yamada H, Hashimoto S, Wakai K, Iso H, Okada E, Fujii R, Hébert JR, Tamakoshi A (2020). Association between dietary inflammatory index and serum C-reactive protein concentrations in the Japan collaborative cohort study. Nagoya J Med Sci.

[34] Na W, Kim M, Sohn C (2018). Dietary inflammatory index and its relationship with high-sensitivity C-reactive protein in Korean: data from the health examinee cohort. J Clin Biochem Nutr.

[35] Jamali R, Raisi M, Matini M, Moravveji A, Omidi A, Amini J. Health related quality of life in irritable bowel syndrome patients, Kashan, Iran: A case control study. Adv Biomed Res. 2015;4(75):1105-15. 10.4103/2277-9175.153902. 10.4103/2277-9175.153902 PMC4386211 25879000

[36] Cassar GE, Youssef GJ, Knowles S, Moulding R, Austin DW (2020). Health-related quality of life in irritable bowel syndrome: a systematic review and meta-analysis. Gastroenterol Nurs.

[37] Wiklund I, Fullerton S, Hawkey C, Jones R, Longstreth G, Mayer E, Peacock RA, Wilson IK, Naesdal J (2003). An irritable bowel syndrome-specific symptom questionnaire: development and validation. Scand J Gastroenterol.

[38] Yildiz A, Kizil E, Yildiz A. Quality of life and psychometric evaluation of patients diagnosed with irritable bowel syndrome: an observational cohort study. Sao Paulo Med J. 2020;138(4):282-6. 10.1590/1516-3180.2019.0527.R1.16042020. 10.1590/1516-3180.2019.0527.R1.16042020 PMC9673838 32556060

[39] Guadagnoli L, Mutlu EA, Doerfler B, Ibrahim A, Brenner D, Taft TH (2019). Food-related quality of life in patients with inflammatory bowel disease and irritable bowel syndrome. Qual Life Res.

[40] Böhn L, Störsrud S, Törnblom H, Bengtsson U, Simrén M (2013). Self-reported food-related gastrointestinal symptoms in IBS are common and associated with more severe symptoms and reduced quality of life. Am J Gastroenterol.

[41] Østgaard H, Hausken T, Gundersen D, El-Salhy M (2012). Diet and effects of diet management on quality of life and symptoms in patients with irritable bowel syndrome. Mol Med Rep.

[42] Philpott H, Gibson P, Thien F (2011). Irritable bowel syndrome-an inflammatory disease involving mast cells. Asia Pacific Allergy.

[43] Coëffier M, Gloro R, Boukhettala N, Aziz M, Lecleire S, Vandaele N, Antonietti M, Savoye G, Bôle-Feysot C, Déchelotte P, Reimund JM, Ducrotté P (2010). Increased proteasome-mediated degradation of occludin in irritable bowel syndrome. Am J Gastroenterol.

[44] Pickett-Blakely O (2014). Obesity and irritable bowel syndrome: a comprehensive review. Gastroenterol Hepatol.

[45] Sobhani SR, Zinab HE. Development of a Computer Assisted Personal Interview (CAPI) tool for food and nutritional surveys using Food Frequency Questionnaire (FFQ) in Iran. J Nutr Sci Diet. 2017;17(8):31-4.

