# Supplementary material for: “Association between dietary inflammatory index (DII) and risk of irritable bowel syndrome: a case-control study”
Source: Nutr J. 2021 Jun 28;20:60. doi: 10.1186/s12937-021-00721-5 (PMC8240213; doi:10.1186/s12937-021-00721-5)
Supplement: Supplementary file 1 — Additional file 1. [file 12937_2021_721_MOESM1_ESM.docx]

| Supplementary Table 1. Distribution of 155 case and 310 control food parameters included in the Dietary Inflammatory Index (DII). | | | | | | |
| --- | --- | --- | --- | --- | --- | --- |
| P value | Mean ± SD | | | | Variables | |
|  | Quartile4  (1.65≤) | Quartile3  (0.11≤ to <1.65) | Quartile2  (-1.49≤ to <0.11) | Quartile1  (<-1.49) |  |  |
| <0.001 | 2420.80±322.16 | 2275.73±349.66 | 2098.27±298.56 | 1896.10±360.78 | Case | Energy (kcal) |
| 0.002 | 2464.10±487.91 | 2400.40±415.39 | 2261.26±401.19 | 2221.55±424.37 | Control |  |
| 0.001 | 74.35±12.70 | 70.50±12.21 | 66.17±11.65 | 63.82±13.32 | Case | Protein (g) |
| 0.385 | 75.74±18.19 | 77.57±18.05 | 75.86±18.18 | 72.86±16.76 | Control |  |
| <0.001 | 354.11±58.18 | 345.58±59.80 | 317.87±58.14 | 293.81±64.43 | Case | Carbohydrate (g) |
| 0.013 | 381.04±79.73 | 373.67±67.86 | 353.81±65.61 | 347.58±74.49 | Control |  |
| <0.001 | 82.93±21.80 | 73.09±23.26 | 66.15±17.27 | 55.65±12.62 | Case | Total fat (g) |
| 0.001 | 75.31±20.43 | 71.18±18.92 | 64.51±15.83 | 65.45±16.33 | Control |  |
| 0.602 | 210.50±57.95 | 205.14±58.30 | 205.78±60.15 | 191.82±60.20 | Case | Cholesterol (mg) |
| 0.708 | 297.36±73.46 | 215.28±76.62 | 219.87±63.17 | 212.41±47.42 | Control |  |
| 0.000 | 18.85±5.24 | 16.06±4.66 | 15.22±4.06 | 13.01±2.91 | Case | Saturated fat (g) |
| 0.139 | 17.02±5.26 | 16.32±4.47 | 15.28±4.37 | 15.81±4.40 | Control |  |
| <0.001 | 28.90±7.28 | 24.97±7.48 | 23.42±6.33 | 18.89±4.50 | Case | MUFA (g) |
| <0.001 | 26.38±6.49 | 24.07±5.95 | 21.63±4.78 | 21.87±5.57 | Control |  |
| <0.001 | 21.94±9.74 | 19.47±8.04 | 17.10±6.51 | 14.20±4.46 | Case | PUFA (g) |
| <0.001 | 19.62±6.65 | 18.00±6.23 | 16.04±5.04 | 15.85±4.93 | Control |  |
| <0.001 | 20.27±9.61 | 17.84±7.78 | 15.52±6.46 | 12.64±4.28 | Case | *n*-6 Fatty acids (g) |
| <0.001 | 17.94±6.46 | 16.46±6.04 | 14.29±4.84 | 14.10±4.74 | Control |  |
| 0.200 | 0.26±0.13 | 0.30±0.18 | 0.26±0.11 | 0.24±0.10 | Case | *n*-3 Fatty acids (g) |
| 0.416 | 0.27±0.16 | 0.27±0.12 | 0.27±0.12 | 0.30±0.14 | Control |  |
| 0.012 | 16.72±3.57 | 16.15±2.89 | 14.99±2.73 | 14.52±3.34 | Case | Fe (mg) |
| 0.789 | 17.49±5.21 | 17.09±3.15 | 16.78±3.93 | 17.18±4.49 | Control |  |
| 0.251 | 203.53±49.92 | 212.32±46.94 | 197.49±40.55 | 221.49±57.49 | Case | Magnesium (mg) |
| 0.001 | 219.97±63.12 | 234.78±54.03 | 236.32±73.36 | 262.26±69.38 | Control |  |
| 0.442 | 7.49±1.96 | 7.26±1.68 | 6.82±1.58 | 7.18±1.75 | Case | Zinc (mg) |
| 0.210 | 7.37±2.30 | 8.04±2.18 | 7.82±2.26 | 8.13±2.16 | Control |  |
| 0.506 | 136.97±45.31 | 143.84±49.29 | 125.34±40.60 | 140.44±70.75 | Case | Selenium (μg) |
| 0.794 | 153.80±54.72 | 160.07±56.17 | 151.71±53.94 | 159.00±74.39 | Control |  |
| 0.064 | 655.31±356.00 | 846.19±493.41 | 846.59±447.46 | 827.37±345.287 | Case | Vitamin A (RE) |
| <0.001 | 761.07±435.50 | 782.48±289.38 | 873.87±412.84 | 1211.86±663.54 | Control |  |
| 0.051 | 190.28±256.53 | 306.03±330.48 | 370.41±399.48 | 296.65±214.53 | Case | β-Carotene (μg) |
| 0.000 | 197.56±120.91 | 206.62±155.92 | 262.84±210.51 | 524.66±496.19 | Control |  |
| 0.180 | 5.14±2.44 | 5.92±3.37 | 5.40±3.22 | 4.42±1.90 | Case | Vitamin E (mg) |
| 0.071 | 5.11±2.88 | 5.62±2.49 | 5.08±2.10 | 5.97±2.66 | Control |  |
| 0.001 | 2.28±0.44 | 2.21±0.39 | 2.03±0.38 | 1.90±0.45 | Case | Thiamin (mg) |
| 0.007 | 2.48±0.49 | 2.41±0.44 | 2.31±0.41 | 2.23±0.50 | Control |  |
| 0.681 | 1.33±0.30 | 1.36±0.36 | 1.27±0.29 | 1.33±0.32 | Case | Riboflavin (mg) |
| 0.528 | 1.51±0.48 | 1.53±0.44 | 1.54±0.46 | 1.61±0.44 | Control |  |
| <0.001 | 24.11±3.94 | 22.82±3.84 | 21.13±4.05 | 19.15±4.14 | Case | Niacin (mg) |
| 0.001 | 24.51±4.85 | 24.31±4.38 | 22.85±4.07 | 21.97±4.59 | Control |  |
| 0.220 | 1.51±0.58 | 1.41±0.56 | 1.24±0.30 | 1.40±0.64 | Case | Vitamin B6 (mg) |
| 0.014 | 1.35±0.53 | 1.51±0.57 | 1.46±0.58 | 1.65±0.56 | Control |  |
| 0.862 | 217.24±74.28 | 223.76±71.01 | 209.87±59.43 | 216.61±43.88 | Case | Folic acid (μg) |
| 0.000 | 220.46±78.56 | 234.20±61.74 | 231.86±65.15 | 268.67±67.35 | Control |  |
| 0.780 | 3.47±2.33 | 3.88±4.02 | 3.19±2.11 | 3.71±2.52 | Case | Vitamin B12 (μg) |
| 0.796 | 4.13±4.07 | 3.79±2.11 | 4.12±3.33 | 4.29±3.23 | Control |  |
| 0.063 | 80.83±27.38 | 92.16±39.24 | 90.92±31.80 | 102.10±43.39 | Case | Vitamin C (mg) |
| <0.001 | 85.46±35.49 | 97.27±41.60 | 104.46±41.48 | 132.80±54.50 | Control |  |
| 0.291 | 0.50±0.48 | 0.74±0.90 | 0.66±0.55 | 0.57±0.56 | Case | Vitamin D (μg) |
| 0.925 | 0.99±1.39 | 0.90±0.94 | 0.88±0.73 | 0.90±0.74 | Control |  |
| 0.518 | 16.45±3.98 | 16.63±3.73 | 15.31±3.10 | 16.43±4.39 | Case | Fiber (g) |
| 0.003 | 17.52±6.63 | 18.06±4.06 | 18.34±4.94 | 20.56±6.37 | Control |  |
| 0.454 | 0.15±0.08 | 0.15±0.11 | 0.18±0.08 | 0.14±0.08 | Case | Caffeine (g) |
| 0.064 | 0.14±0.08 | 0.15±0.08 | 0.13±0.06 | 0.17±0.10 | Control |  |
| 0.587 | 0.76±1.63 | 0.57±0.84 | 0.82±1.08 | 1.04±1.72 | Case | Garlic (g) |
| 0.214 | 1.32±2.77 | 1.00±1.22 | 1.25±1.62 | 1.63±2.00 | Control |  |
| 0.204 | 15.88±14.19 | 13.51±17.21 | 11.35±14.64 | 9.04±9.81 | Case | Onion(g) |
| 0.868 | 17.14±23.22 | 16.93±16.92 | 17.70±24.41 | 20.06±37.32 | Control |  |
| 0.742 | 0.04±0.17 | 0.04±0.11 | 0.04±0.09 | 0.08±0.20 | Case | Ginger (g) |
| <0.001 | 0.002±0.006 | 0.02±0.06 | 0.08±0.35 | 0.25±0.60 | Control |  |
| 0.300 | 0.00±0.00 | 0.01±0.07 | 0.00±0.00 | 0.01±0.02 | Case | Saffron (g) |
| 0.001 | 0.00±0.01 | 0.00±0.01 | 0.00±0.02 | 0.01±0.02 | Control |  |
| <0.001 | 0.29±0.35 | 0.23±0.31 | 0.35±0.49 | 1.03±1.17 | Case | Black Pepper (g) |
| <0.001 | 0.24±0.35 | 0.51±0.57 | 0.52±0.50 | 0.86±0.98 | Control |  |
| 0.055 | 10.86±29.74 | 23.05±44.32 | 23.38±40.12 | 118.36±407.76 | Case | Thyme (mg) |
| 0.001 | 7.84±27.02 | 29.65±73.21 | 34.61±81.35 | 119.56±332.19 | Control |  |
| 0.525 | 0.56±3.39 | 2.57±15.45 | 0.00±0.00 | 0.30±1.58 | Case | Rosemary (mg) |
| 0.153 | 0.70±3.85 | 0.32±2.09 | 0.19±1.24 | 10.25±63.53 | Control |  |
| <0.001 | 613.17±504.42 | 848.63±810.30 | 971.93±744.42 | 1727.51±1464.92 | Case | turmeric(mg) |
| <0.001 | 619.93±394.22 | 805.34±521.32 | 1058.45±1018.81 | 1534.52±1295.07 | Control |  |
| 0.416 | 735.36±413.23 | 707.75±560.35 | 865.71±414.86 | 675.55±410.52 | Case | Black tea (g) |
| 0.114 | 646.24±397.63 | 691.63±402.90 | 619.93±281.18 | 745.14±355.81 | Control |  |
| PUFA, polyunsaturated fatty acids; MUFA, monounsaturated fatty acids.  ^(a)^ The One-way analysis of variance (ANOVA) was used for comparison of the variables between DII quartiles. | | | | | | |
